# Flasking

**Published:** 1906-12

**Authors:** Stewart J. Spence

**Affiliations:** Chattanooga, Tenn.


### FLASKING.

STEWART J. SPENCE, CHATTANOOGA, TENN.

Having arranged the teeth on the wax trial-plate and tried them on the living subject, the most scientific and pleasant part of making a prosthetic denture is done, and the remainder is comparatively routine and drudgery work, requiring patience rather than skill. Still there are some things may be said on the subject not already known to all readers, especially with regard to the shrinkage of vulcanite.

During the process of cooling, vulcanized rubber will contract about two per cent. of its diameter, if not prevented from doing so. Anything that will hold on to the vulcanite will more or less prevent this shrinkage. Of course the plaster investment does hold on to it to some extent, especially if the surface of the plate be rough or if there be many air pits in the investment, into which the rubber has protruded. These protruding particles prevent to some extent the contraction which takes place in the area of vulcanite (that is, in the length and breadth of the plate), and perhaps also to some extent in its thickness, if they are plentiful. But my experiments have demonstrated very satisfactorily to myself that an investment of plaster of Paris is never sufficiently solid to prevent *all* areal contraction, and there is no doubt at all that vulcanite plates always contract when invested with it. When this contraction is even, so that one side of the plate contracts as much as the other, there is generally no evil result, but on the contrary, it is this very contraction which, by counteracting the expansion of the plaster of Paris impression and model, saves many a plate from being an utter misfit. Therefore if Spence's Nonexpansive Plaster has not been used for the model and impression, or at least for the model, it is better not to use it for the investment, because its good quality of solidity will, by preventing the areal contraction of the vulcanite, maintain the plate at the over-large dimensions given it by the model.

But areal contraction does not always proceed evenly. Often one side of a plate contracts apparently at the expense of the other (perhaps because less firmly held by the plaster of Paris investment), and in these cases there is warpage and misfit. The harder the investment the less is the danger of said warpage; to avoid which I would advise those who use plaster of Paris to let their investments harden over night before vulcanizing were it not that such hardening tends to lessen the even contraction which, as beforesaid, counteracts the expansion of the model, thus presenting only a choice of evils.

But besides this areal contraction here is that which proceeds through the thickness of the plate,—vertical contraction. At first sight it might seem that so small a matter as two per cent. of, say, one-fourh inch, could not largely affect the adhesion of a plate; but "every little counts" in this matter, and some plates are as much as a half inch thick in places. Now, as the investment does not appreciably prevent this contraction of thickness, especially if the model be coated (as it certainly ought to be) with tin foil, it proceeds to nearly the full two per cent.; and as the contraction of the plate's thick parts (that is, those over the ridge) tends to throw pressure on the palate, an undesirable result is produced.

The dentist should bear in mind this fact of the contraction of rubber while waxing up the piece. He should make it of as even a thickness all over as may be; because equal contraction does no evil. If he uses plumpers, he should make them of the wing shape, not by making a thick mass of vulcanite; and he should be watchful to avoid that undue thickness of wax which is apt to occur on either side of the median line of the palate. In nature the palate is not a rounded arch, like the Roman arch, as it usually appears in vulcanite plates (lingual surface), but is comparatively flat at the roof; indeed, sometimes higher on either side of the median line than at it. In such cases the plate may be of correct thickness (about one-sixteenth inch) on said line, while being three-sixteenth on either side; thus giving one-eighth in which contraction counts for bad.

This undue thickening is especially apt to occur when the blow-pipe is used to smooth the wax.

Another thing to be watched against when the blow-pipe is used, is the little dimple in the wax which it leaves. These dimples, by filling with plaster points, too small to be readily observed by the eye, result in pits of plaster in the vulcanite plate which require scraping or grinding to obliterate.

The writer constantly uses the blow-pipe for smoothing. It is especially helpful on the buccal and labial surfaces. The flowing wax fills correctly the interdental spaces, which, when the wax is shaped only by carving, are apt to be cut out deeply. This is a very common error. In nature the V-shaped interdental gingavæ are on about the level of the teeth, and ought be so in prosthetic dentures. To say that the other method prevents a feather edge that will eventually curl up from the porcelain tooth and permit detribus to insert itself, is not good argument, for the edge of a pink rubber on celluloid gum ought never to be left this thin; and need not be. To give the wax the depressions between the roots, the writer heats his wax spatula and lays it on the wax gums so as to melt out a hollow. A puff of the blow-pipe smooths this, and the rest, that is, the trimming of the gum margin, is done by the carving tool after vulcanization. Those who have the time, and are ambitious of very artistic results, will do well to study Geo. H. Wilson's method of forming the festoons in wax by aid of a string, and coating them with tin foil, as published some time ago in the Dental Summary.

I have never been persuaded that prosthetic rugæ pay for the trouble of making them and the after labor of keeping them clean.

In purchasing flasks, it is well to get large ones,—as wide as the vulcanizer will receive. Many flasks are too small, and necessitate great waste of time and effort in trimming models to such shape that they will go in the flask and permit separation without fracture. Plaster is easier cut by plate shears than by knife.

To avoid fracture, often a coat of ordinary separating fluid, such as soap in solution, is insufficient. A film of melted wax flowed over the surface suffices.

